# Anterior release & posterior spinal fusion vs. posterior-only fusion in AIS patients with large magnitude thoracic curves

**DOI:** 10.1007/s00590-025-04598-6

**Published:** 2026-02-04

**Authors:** Anabelle Permutt, Zacharia Silk, Julian Leong, Hilali Noordeen, Jan Lehovsky, Sean Molloy, Alexander Gibson, Roozbeh Shafafy

**Affiliations:** 1 University College London Medical School, London, UK; 2 Royal National Orthopaedic Hospital, Stanmore, UK

**Keywords:** Adolescent idiopathic scoliosis, Anterior, Posterior

## Abstract

**Purpose:**

The surgical management of severe adolescent idiopathic scoliosis (AIS) remains a subject of ongoing discussion among spine-deformity surgeons. While there has been a prevailing shift towards posterior-only fusion (PF) techniques, anterior release with posterior fusion (ARPF) may still be valuable for the correction of large or stiff curves. This study compared the clinical and radiological outcomes of PF and ARPF in a high-volume spinal deformity unit.

**Methods:**

Patients aged 10–18 years with AIS and a major thoracic curve ≥ 70^°^ (Lenke Types 1–4), who underwent PF or ARPF between 2010 and 2019, with ≥ 2 years of follow-up were included. Correction index formed the primary outcome, complemented by several secondary radiological and clinical measures assessed preoperatively, at first-erect radiograph and final follow-up.

**Results:**

Eighty-nine patients were included (PF = 51; ARPF = 38). Baseline characteristics were similar except ARPF patients were younger (13.7 vs 1.4.8, *p* = 0.001) and less skeletally mature (Risser 0–2: 60.5% vs 37.3%). Baseline Cobb angle was comparable (*p* = 0.0634), but ARPF patients had stiffer curves (*p* = 0.0257), reduced flexibility (*p* = 0.0408), and achieved a significantly higher CI at final follow-up (248.2% vs. 168.1%, *p* = 0.0024). Operative time, blood loss and length of stay were greater for ARPF.

**Conclusions:**

ARPF offers a corrective advantage for patients with stiffer curves and lower skeletal maturity, who are at risk of developing crankshaft phenomena. However, the increased operative morbidity supports PF alone as sufficient for most patients. These results highlight the importance of tailoring surgical strategy to curve characteristics and skeletal maturity to optimise correction and minimise risk.

**Supplementary Information:**

The online version contains supplementary material available at 10.1007/s00590-025-04598-6.

## Introduction

The optimal management of large-magnitude curves (≥ 70°) in patients with adolescent idiopathic scoliosis (AIS) remains a topic of ongoing debate among spine deformity surgeons. These curves often exhibit increased rigidity, posing challenges to conventional posterior-based fusion techniques and raising concerns regarding blood loss, operative time, and complications. While the primary objective of surgical intervention is to prevent the anticipated progression of deformity, there is a strong emphasis on meeting the elevated cosmetic and functional expectations of this patient population. Consequently, achieving a well-balanced, functional, and aesthetically acceptable correction in both coronal and sagittal planes holds significant importance [1].

**Fig. 1:**
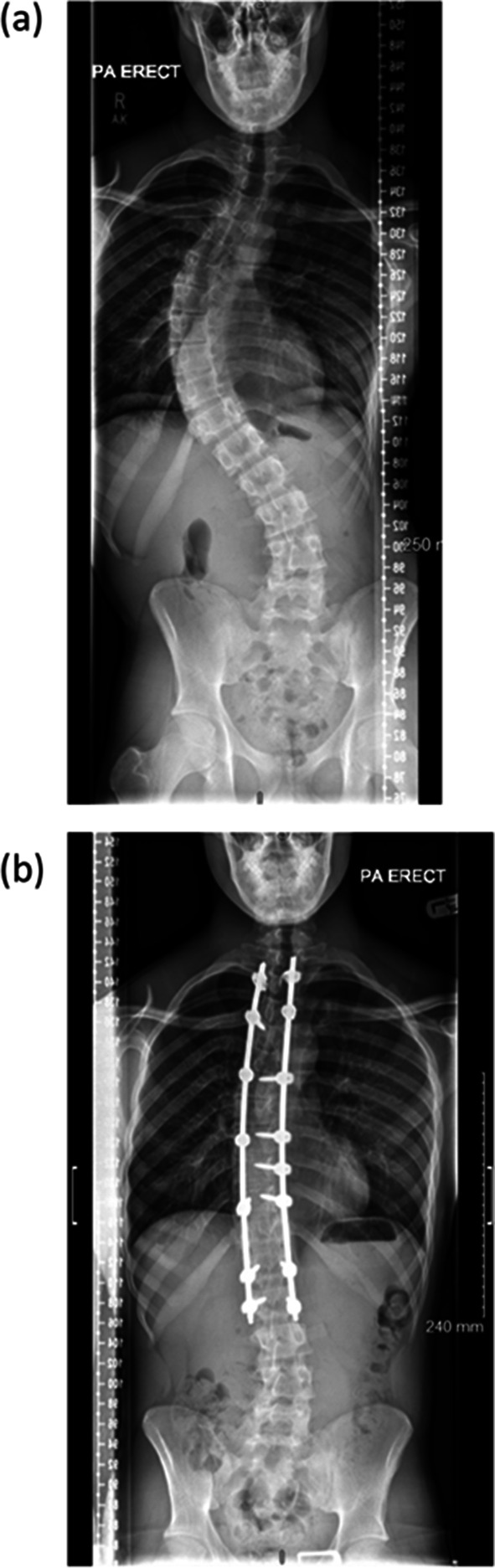
Radiographic images before and after the posterior-only instrumented fusion. **a** Preoperative standing antero-posterior radiograph. **b** postoperative standing antero-posterior radiograph

Instrumented posterior-only fusion (PF) remains the gold standard technique, dating back to Harrington’s seminal report in 1962 [1] (Fig. 1). Modern implant systems, largely evolved from the Cotrel-Dubousset design [1], enable three-dimensional corrective force across multiple levels of the spine and have long surpassed the capabilities of the prior surgical implants [2]. Yet continued anterior-column elongation in patients with significant anticipated growth may still lead to the development of the crankshaft phenomenon and progressive deformity, although this is less likely as modern pedicle screws span both anterior and posterior columns, providing greater construct stiffness [3, 4].

**Fig. 2:**
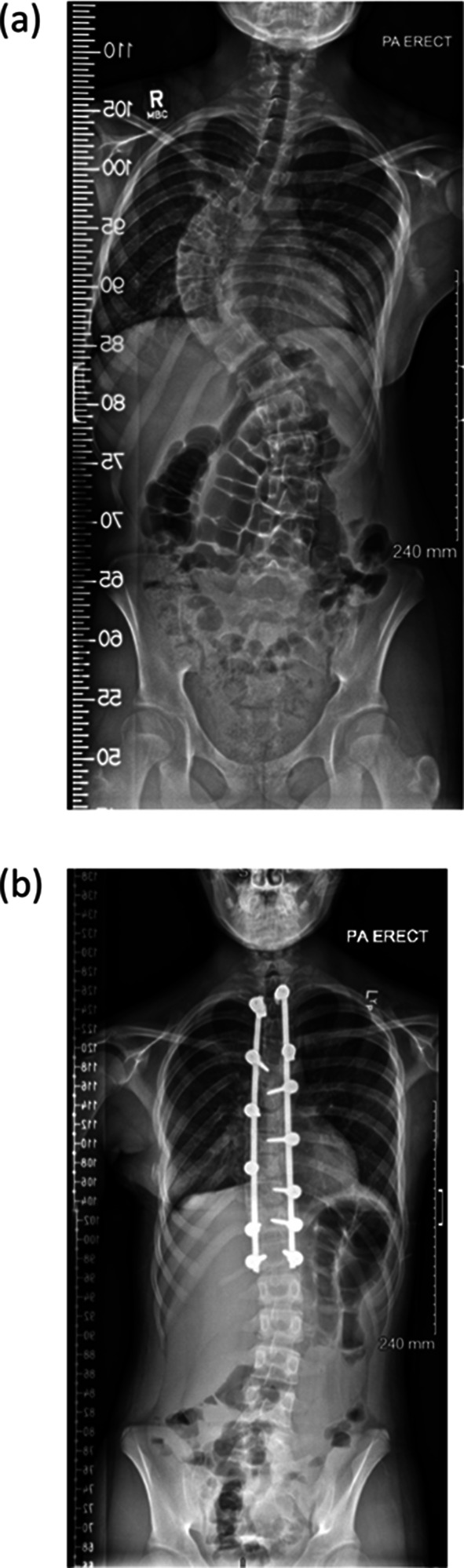
Radiographic images before and after the combined anterior release and posterior instrumented fusion. **a** Preoperative standing antero-posterior radiograph. **b** Standing antero-posterior radiograph after both stages (combined anterior release and posterior instrumented fusion)

In selected cases, particularly in patients with large and rigid curves, anterior release combined with posterior fusion (ARPF) may be more appropriate (Fig. 2). As an anterior column shortening procedure, this provides space for the spine to derotate into by removing its principal constraint, the intervertebral disc [5]. ARPF has demonstrated significant advantages, including superior radiographic correction [6] and greater patient satisfaction [3], and surgeons may also favour this approach for patients with severe coronal imbalance and razorback deformity, which may be further managed with a costoplasty [7]. However, ARPF is associated with inherent drawbacks, including an initial negative impact on pulmonary function [8] and increased surgical invasiveness and risk [9].

This observational study compares the radiographic and clinical outcomes of patients with AIS treated with either PF alone or ARPF at a high-volume spinal deformity centre. This study distinguishes itself by focusing specifically on the management of patients presenting with large-magnitude curves (≥ 70°).

## Materials and methods

### Institutional review board approval

The study protocol received prospective approval from the institution’s research ethics board before commencing data collection (IRB: SE22.02).

### Patients

**Fig. 3:**
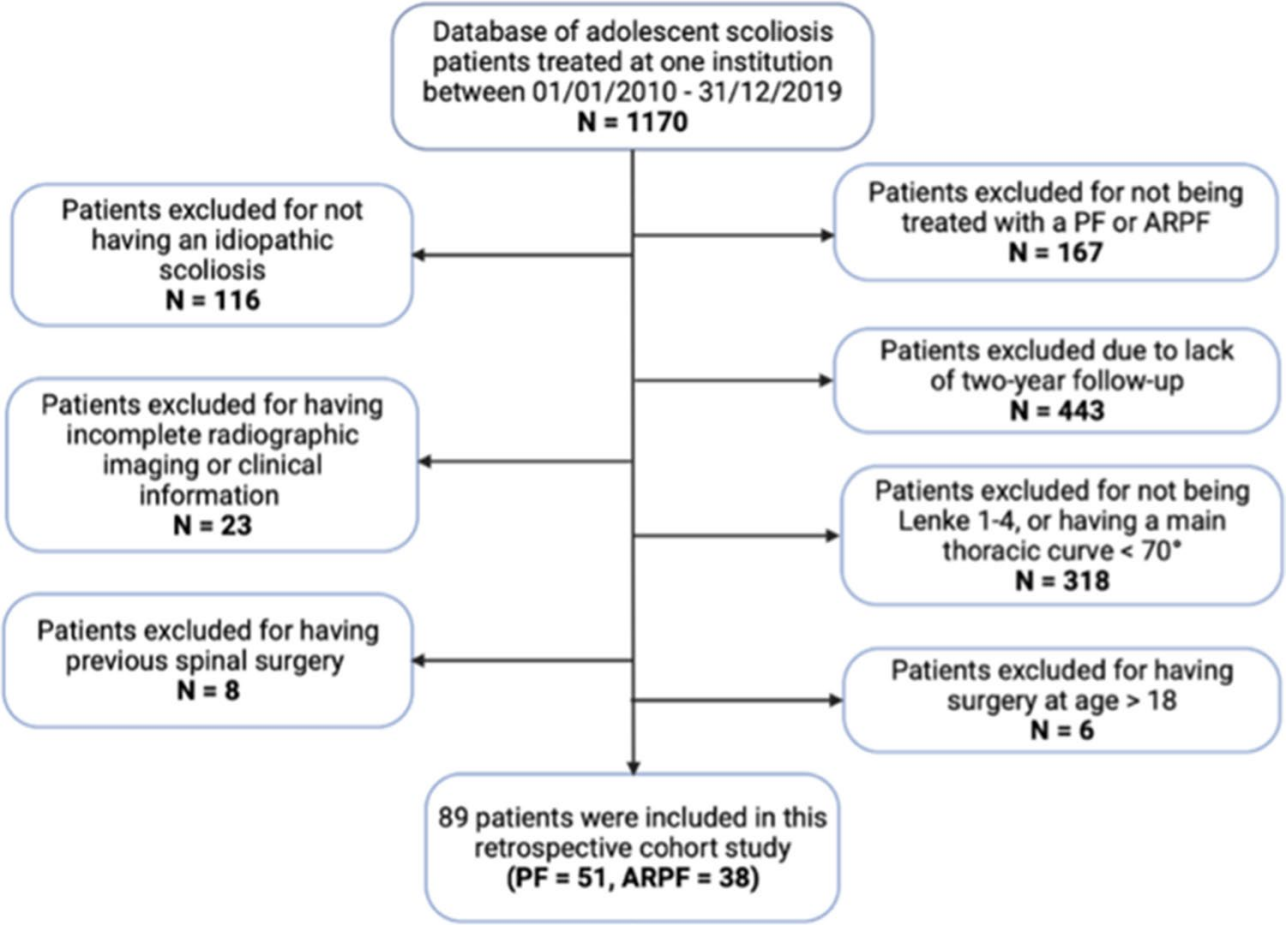
Flow diagram of study participants

Between 1st January 2010 and 31st December 2019, a total of 1170 adolescent patients with idiopathic scoliosis underwent surgical correction by five experienced surgeons at a high-volume spinal deformity unit. A meticulous application of the following inclusion and exclusion criteria identified 89 patients suited for this study (Fig. 3).

Inclusion criteria:

- Diagnosis: AIS
- Age at surgery: 10–18 year
- Curve types: Lenke 1–4 (i.e. all include a structural main thoracic (MT) curve)
- Curve size: MT Cobb angle ≥ 70°
- Treatment: PF or ARPF
- Follow-up: minimum two-years of clinical and radiological follow-up. A four-month grace period was permitted for radiographic follow up in cases where the clinical follow up was more than two-years

Exclusion criteria

- Prior spinal surgery
- Incomplete radiological and/or clinical records
- Patients with non-idiopathic or early-onset causes of scoliosis
- Surgical treatment with anterior instrumented fusion

Patients were initially screened for enrolment by AP. Borderline cases with major curves of 70° ± 5° underwent a second assessment by a second assessor (ZS) to determine suitability for inclusion. Any discrepancies were resolved by a third assessor (RS).

### Intervention group

Both cohorts underwent posterior instrumented correction and fusion for their scoliosis. Of the 89 patients included in this study, 51 were treated with PF alone. This involved an open midline sub-periosteal approach with freehand and/or 3D navigation-based insertion of pedicle screws and/or hooks, with one case involving more extensive releases (Ponte-osteotomies), and one requiring supplementary instrumentation (sublaminar bands). The upper and lower instrumented levels, implant density, rod shape and type were also noted.

### Comparison group

The comparison group (n = 38) underwent ARPF. An open thoracotomy approach was used for anterior release and additional procedures (e.g. rib head resection or anterior costoplasty) were recorded. Complications related to the surgical approach and chest drains were noted. Staged PF, following initial perioperative optimisation, was standard practice, with a mean wait of 5.7 ± 4.2 days between procedures.

### Radiological outcomes

**Fig. 4:**
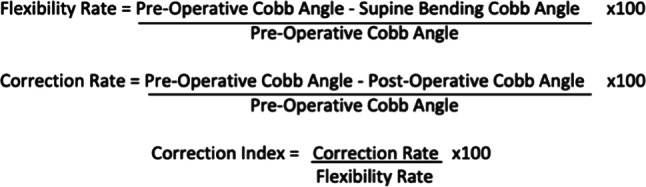
Calculations for flexibility rate, correction rate and correction index

Radiographs were assessed using the institution’s Picture Archiving and Communication System (PACS) (McKesson Enterprise Medical Imaging). The Cobb method was applied to determine the magnitudes of proximal thoracic, main thoracic (MT) and thoracolumbar/lumbar curves on preoperative posteroanterior and supine lateral bending (SLB) radiographs, in addition to postoperative first erect (FER) and final follow-up radiographs. Preoperative skeletal maturity markers were also noted. Flexibility rate (FR), correction rate (CR) and correction index (CI) were calculated (Fig. 4).

Thoracic kyphosis (TK) and sagittal vertical axis (SVA) were evaluated with lateral whole spine radiographs. Coronal balance (CB), clavicle angle (CA) and trunk shift (TS) were assessed. Curves were categorised according to the Lenke classification[10].

### Clinical outcomes

Electronic medical records provided clinical information relating to aetiology and onset of scoliosis, procedure, admission and discharge dates, length of stay (LOS), operative time(s) (OT), estimated blood loss (EBL), complications and implant details. Intraoperative neuromonitoring logs and surgical site infection surveillance records were obtained.

### Primary and secondary outcomes

The primary outcome of this study was the CI—a standardised measure of the correction achieved after controlling for initial curve magnitude and flexibility [11]. Secondary outcomes included response of the sagittal profile to treatment, radiological shoulder balance (CA); waist symmetry (TS), complications and unplanned return to theatre.

### Statistical analyses

Prism 9 Software (GraphPad Software, Boston, Massachusetts, USA, www.graphpad.com) was used for statistical analysis. A sample size calculation confirmed a minimum of 33 patients in each cohort would support a significance value of 5% at a power of 90%. Continuous variables were presented as mean difference ± standard deviation, categorical variables as percentages. The unpaired t-test with the Welch’s correction and χ^2^ test were used to compare groups, while the paired t-test was used to compare outcomes within groups. *P *values < 0.05 were deemed significant.

## Results

### Baseline demographics

Baseline characteristics are noted in Table 1. A statistically significant difference was observed in mean age, with the ARPF group being younger in age (13.7 vs. 14.8 years; *p* = 0.0011). Although not statistically significant, patients in the ARPF group were clinically less skeletally mature based on their Risser Sign. While the mean preoperative MT cobb angle was radiologically and statistically similar between groups, the ARPF cohort exhibited significantly less flexibility on SLB radiographs (37.2% vs. 30.9%; *p* = 0.0408). Despite the inclusion criteria specifying a thoracic Cobb angle ≥ 70°, the mean Cobb angles in both PF and ARPF groups were ≥ 80°.

**Table 1:** Basic characteristics of the patients in the combined anterior release and posterior fusion group versus posterior-only fusion group

	PF, n = 51	ARPF, n = 38	P
Age, years	14.8 ± 1.5	13.7 ± 1.4	0.0011*
Sex, n (%)			0.2066†
Male	15 (29.4%)	6 (15.8%)	
Female	36 (70.6%)	32 (84.2%)	
Risser sgn, n (%)			0.093 ~
0,1,2	19 (37.3%)	23 (60.5%)	
3	7 (13.7%)	3 (7.9%)	
4,5	25 (49.0%)	12 (31.6%)	
Triradiate cartilages, n (%)			0.6473†
Open	2 (3.9%)	3 (7.9%)	
Closed	49 (96.1%)	35 (92.1%)	
Lenke type, n (%)			0.997 ~
1	40 (78.4%)	30 (78.9%)	
2	7 (13.7%)	5 (13.2%)	
3	4 (7.8%)	3 (7.9%)	
Lenke lumbar modifier, n (%)			0.1445 ~
A	27 (52.9%)	25 (65.8%)	
B	10 (19.6%)	9 (23.7%)	
C	14 (27.5%)	4 (10.5%)	
ASA, n (%)			0.1242 ~
1	40 (78.4%)	26 (68.4%)	
2	9 (17.6%)	13 (34.2%)	
3	2 (3.9%)	0 (0%)	
Number of levels fused	12.0 ± 1.9	12.1 ± 1.6	0.7588*
Screw density	62.4 ± 10.3	60.3 ± 6.4	0.2453*
Follow-up, months	58.2 ± 25.7	58.5 ± 26.9	0.9485*
Main thoracic curve pre-op, °	80.0 ± 11.1	84.2 ± 9.7	0.0634*
Main thoracic bending cobb, °	50.9 ± 16.3	58.5 ± 15.1	0.0257*
Main thoracic flexibility rate, %	37.2 ± 13.3	30.9 ± 14.8	0.0408*

*Welch’s t test

† Fisher’s exact test.

~ Chi-squared test

### Primary outcome

The CR for both groups were similar at all time-points, but when preoperative curve stiffness was controlled for, the overall CI was significantly better in the ARPF group at both FER (*P* = 0.0029) and Final Follow-Up (*P* = 0.0024). In both groups, a loss of curve correction was observed between FER and Final Follow-Up radiographs, but this loss was only statistically significant in the ARPF group (*P* = 0.0479). This is summarised in Table 2.

**Table 2:** Radiographic assessment in the coronal and sagittal profiles

	PF, n = 51	ARPF, n = 38	P*
*Main thoracic cobb, °*			
Preoperative	80.0 ± 11.1	84.2 ± 9.7	0.0634
FER	33.8 ± 12.4	28.2 ± 8.9	0.0149
Final follow-up	35.6 ± 14.9	32.1 ± 11.5	0.2139
*Main thoracic correction rate, %*			
FER	70.8 ± 8.3	73.5 ± 9.5	0.168
Final follow-up	56.0 ± 15.0	61.8 ± 13.7	0.0654
Main thoracic correction index, %			
FER	176.3 ± 70.0	276.4 ± 185.8	0.0029
Final follow-up	168.1 ± 69.1	248.2 ± 142.2	0.0024
**P†**	0.1142	0.0497	
Coronal balance, mm			
Preoperative	8.2 ± 23.0	14.2 ± 19.3	0.1915
FER	− 0.3 ± 22.2	2.4 ± 19.5	0.5496
Final follow-up	− 0.5 ± 15.1	− 3.4 ± 17.0	0.4058
Clavicle angle, °			
Preoperative	− 1.1 ± 3.0	− 1.5 ± 2.5	0.5653
FER	3.3 ± 3.3	2.7 ± 3.3	0.3853
Final follow-up	1.5 ± 2.8	1.7 ± 2.0	0.7548
*Trunk shift, mm*			
Preoperative	29.4 ± 21.6	34.5 ± 20.4	0.2558
FER	− 3.1 ± 14.3	− 9.2 ± 15.1	0.0581
Final follow-up	0.1 ± 16.8	− 9.0 ± 14.5	0.0077
Thoracic kyphosis, °			
Preoperative	24.9 ± 13.1	30.7 ± 13.4	0.0426
FER	17.1 ± 8.5	22.2 ± 7.8	0.0043
Final follow-up	18.6 ± 9.5	25.2 ± 7.8	0.0006
*SVA, mm*			
Preoperative	5.0 ± 36.3	7.7 ± 34.2	0.7227
FER	28.8 ± 41.2	29.4 ± 32.3	0.9402
Final follow-up	− 19.2 ± 31.6	− 11.7 ± 30.4	0.2582

* Welch’s t test

† Paired t test.

Coronal Balance: +  = C7PL right of CSVL,—= C7PL left of CSVL.

Clavicle angle: +  = left shoulder higher than right,—= right shoulder higher than left.

Trunk shift: +  = trunk is shifted to right of CSVL,—= trunk is shifted to left of CSVL.

SVA: + result denotes C7PL falling infront of the posterosuperior corner of S1.—result denotes C7PL falling behind the posterosuperior corner of S1.

### Secondary outcomes

#### Radiographic outcomes

Both PF and ARPF similarly restored and maintained CB within 20mm of the CSVL at FER (*p* = 0.5496) and Final Follow-Up (*p* = 0.4058). CA was also similar at all timepoints and there appears to be a progressive improvement observed between FER and Final Follow-Up. Both groups demonstrated statistically similar (*p* = 0.2558), but radiologically significant deviations in preoperative TS (i.e. > 20mm). Restoration of TS was statistically better following PF rather than ARPF (*p* = 0.0077) at Final Follow-Up, but the mean magnitude of shift away from the CSVL remained within clinically acceptable limits (< 20mm) in both cohorts. These results are summarised in Table 2.

Regarding the sagittal profile, TK was greater in the ARPF group (*p* = 0.0426). A reduction in the overall TK following surgery compared to baseline was noted in both groups, yet ARPF was statistically more likely to produce a better TK at FER (*p* = 0.0043) and Final Follow-Up (*p* = 0.0006). Global balance of the spine represented by the SVA was similar between groups at all time-points and within clinically acceptable limits (≤ 40mm) (Table 2).

Table 3 describes differences between preoperative and both postoperative time-points. Surgery resulted in significant improvements in both CB and TS. In keeping with expected results, the CA was initially worse at FER and improved by Final Follow-Up to within clinically acceptable limits. With respect to TK, surgery resulted in a hypokyphosis in both PF (*p* < 0.0001) and ARPF (*p* = 0.0006) at FER. This remained similar in the PF group, however there was a statistically significant increase in kyphosis between FER and Final Follow-Up in the ARPF group (*p* = 0.0123). Although SVA became more positive in both groups following surgery, over time there was a spontaneous change towards negative SVA at Final Follow-Up in both groups (*p* < 0.0001).

**Table 3:** Changes in secondary radiographic parameters over time

	PF	ARPF				
	Pre-op	Follow-up	P*	Pre-op	Follow-up	P*
*Thoracic kyphosis, °*						
Pre-operative to FER	24.9 ± 13.1	17.1 ± 8.5	< 0.0001	30.7 ± 13.4	22.2 ± 7.8	0.0006
Pre-operative to final follow-up	24.9 ± 13.1	18.6 ± 9.5	0.0024	30.7 ± 13.4	25.2 ± 7.8	0.0244
*SVA, mm*						
Pre-operative to FER	5.0 ± 36.3	28.8 ± 41.2	< 0.0001	7.7 ± 34.2	29.4 ± 32.3	< 0.0001
Pre-operative to final follow-up	5.0 ± 36.3	− 19.2 ± 31.6	0.0064	7.7 ± 34.2	− 11.7 ± 30.4	0.0046
*Coronal balance, mm*						
Pre-operative to FER	8.2 ± 23.0	− 0.3 ± 22.2	0.0202	14.2 ± 19.3	2.4 ± 19.5	0.001
Pre-operative to final follow-up	8.2 ± 23.0	− 0.5 ± 15.1	0.0028	14.2 ± 19.3	− 3.4 ± 17.0	< 0.0001
*Clavicle angle, °*						
Pre-operative to FER	− 1.1 ± 3.0	3.3 ± 3.3	< 0.0001	− 1.5 ± 2.5	2.7 ± 3.3	< 0.0001
Pre-operative to final follow-up	− 1.1 ± 3.0	1.5 ± 2.8	< 0.0001	− 1.5 ± 2.5	1.7 ± 2.0	< 0.0001
*Trunk shift, mm*						
Pre-operative to FER	29.4 ± 21.6	− 3.2 ± 14.3	< 0.0001	34.5 ± 20.4	− 9.2 ± 15.1	< 0.0001
Pre-operative to final follow-up	29.4 ± 21.6	0.1 ± 16.8	< 0.0001	34.5 ± 20.4	− 9.0 ± 14.5	< 0.0001

* Paired t test

SVA: +  = C7PL falling infront of the posterosuperior corner of S1.—= C7PL falling infront of the posterosuperior corner of S1.

Coronal balance: +  = C7PL right of CSVL,—= C7PL left of CSVL.

Clavicle angle: +  = left shoulder higher than right,—= right shoulder higher than left.

Trunk shift: +  = trunk is shifted to right of CSVL,—= trunk is shifted to left of CSVL.

### Clinical outcomes

#### Perioperative results

The mean total operating time (OT) and estimated blood loss (EBL) for PF (in both the PF group, and the posterior stage of the ARPF group) was 244.4 ± 165 min and 708.4 ± 452.5mls, respectively. First stage anterior release in the ARPF cohort resulted in an additional mean OT of 155.3 ± 43.6 min, and EBL of 228.1 ± 137.2 mls. The total combined OT and EBL for the ARPF group were 398.9 ± 86.3 min and 965.3 ± 446.3 mls, respectively. The ARPF group exhibited significantly larger OT, EBL, and LOS than the PF group (Table SI—Supplementary Information).

#### Complications

The overall complication rate amongst the study population was 15.7% (14/89 patients). ARPF resulted in a higher rate of complications when compared to PF (26.3% vs 7.8%) however did not reach statistical significance (*p* = 0.8268). Pulmonary complications and metalwork failure (i.e. loosening, rod failure, pseudoarthrosis) were more common in the ARPF group.

## Discussion

To our knowledge, this study represents the largest comparative analysis of posterior-only fusion (PF) versus combined anterior release with posterior fusion (ARPF) in AIS patients with large-magnitude thoracic curves (Lenke 1–4; Cobb ≥ 70°).

Our findings demonstrate that both ARPF and PF achieved substantial coronal correction, consistent with the literature. When preoperative stiffness was accounted for using the correction index (CI), ARPF demonstrated a significantly greater capacity for correction at first-erect and at final follow-up. Notably, our ARPF cohort exhibited stiffer curves, and greater skeletal immaturity, supporting the role of a targeted anterior release to unlock correction in rigid deformities, especially when the potential for crankshaft may be a concern.

Contemporary posterior segmental instrumentation has diminished the routine need for anterior procedures in many AIS patterns [1–4]. Prior comparative work has suggested only modest incremental benefit from adding an anterior release [12] and highlighted a negative impact on pulmonary function [22–24]. Variation in release technique and patient selection likely explain discrepancies with other series [12].

Across several parameters, we observed some loss of correction between first-erect and final follow-up radiographs, reaching statistical significance in the ARPF group. Given the younger and lower skeletal maturity in the ARPF group, continued skeletal growth, particularly of the anterior column, may contribute to progressive loss of correction and crankshaft [13], despite modern pedicle-screw constructs spanning both columns [4], and underscores the importance of appropriate counselling and managing expectations.

Restoration of physiological thoracic kyphosis (TK) remains a priority for energy-efficient gait and global balance [14]. In AIS, this relies on either anterior column shortening, posterior column lengthening, or a combination of the two techniques. Betz et al. compared PF to ARPF in this context and found correction of TK to be 40% and 92%, respectively [15]. This contrasts with our findings, where both techniques produced hypokyphosis relative to baseline at first-erect. While ARPF maintained comparatively greater TK at both postoperative timepoints, neither approach “restored” TK beyond baseline values, contrasting with earlier studies reporting larger kyphosis gains after anterior release [15]. Nevertheless, TK values for both groups remained within an accepted normal range of 10–40° at follow-up [16], and global alignment (SVA) was comparable and clinically acceptable throughout.

Previous literature has highlighted a clear discord between surgeon and patient priorities in the management of AIS. Visible improvements in cosmetic deformity (e.g. shoulder balance (CB) and trunk symmetry (TS)) often correlate more closely with patient satisfaction than angular correction [17, 18]. In both cohorts, coronal balance significantly improved after surgery and was comparable between groups. Although TS was statistically better corrected amongst the PF group, this was deemed clinically insignificant, as CB or TS within 20mm of the CSVL falls within acceptable limits [19]. Clavicle angle—a surrogate for shoulder balance—was also comparable between groups. While ipsilateral shoulder elevation was observed in both cohorts, this improved into a clinically acceptable range over time, mirroring prior reports of spontaneous shoulder levelling [20, 21]. Therefore, it seems reasonable to conclude that either surgical strategy can meet patient and surgeon priorities for frontal-plane appearance when appropriately selected.

The main disadvantages of ARPF are the well-recognised decline in pulmonary function (vital capacity and forced expiratory volume) and associated complications [1, 12, 22–24], as well as increased OT and LOS [25]. Coe et al. [24] reported pulmonary complications as the most common adverse event in patients undergoing ARPF, with a prevalence of 3.5%. This finding was similarly reflected in our study, with pulmonary complications accounting for 7.9% of cases. Coe et al. [24] also reported wound infection in 1.35% of PF cases; our study found a similar rate (1.96%), although the most common complication of the PF group was intraoperative neuromonitoring alerts (7.8%). Only 2.5% of these events resulted in neurological injury. In this study, the two groups were comparable with respect to return-to-theatre rates and overall complication profiles. Nevertheless, the significantly shorter OT, LOS and EBL observed in the PF group are clear advantages, rendering PF the more economically favourable option and less burdensome for both hospitals and patients.

While limitations exist, the key strengths of this study include a focused indication (≥ 70° thoracic curves) and the use of CI as a stiffness-adjusted primary endpoint, which reduces confounding by baseline rigidity [11]. Limitations include the retrospective design, potential selection bias towards younger/less mature patients for ARPF, and reliance on supine lateral bending radiographs to estimate flexibility, where patient cooperation and effort reduces reliability compared to fulcrum bending or traction films [9]. Absence of pulmonary function testing and patient-reported outcomes limits the ability to weigh radiographic gains against respiratory impact and patient satisfaction. Despite these limitations, this study still provides a valuable and meaningful insight into the radiological and clinical outcomes of the two techniques.

Prospective studies incorporating standardised flexibility assessment (e.g. fulcrum-bending/traction), pulmonary function measurement and validated patient-reported outcomes are needed to better define the value proposition of ARPF more precisely. Stratified analyses by skeletal maturity and curve rigidity could yield practical decision algorithms for selecting PF versus ARPF in large-magnitude curves affecting patients with AIS.

## Conclusion

For large, stiff thoracic curves in skeletally immature patients with AIS—those at greatest risk of under correction and crankshaft, an anterior release offers a tangible corrective advantage after adjusting for stiffness. However, this must be balanced against the additional risks involved with increased surgical invasiveness, pulmonary risk and resource utilisation. In contrast, for large but flexible curves, modern PF alone remains preferable given similar radiological outcomes, shorter operations, lower blood loss, and shorter hospitalisation.

## Supplementary Information

Below is the link to the electronic supplementary material.

Supplementary Material 1.

## Data Availability

No datasets were generated or analysed during the current study.
