# Supplementary material for: Anterior release & posterior spinal fusion vs. posterior-only fusion in AIS patients with large magnitude thoracic curves
Source: Eur J Orthop Surg Traumatol. 2026 Feb 4;36(1):96. doi: 10.1007/s00590-025-04598-6 (PMC12872680; doi:10.1007/s00590-025-04598-6)
Supplement: Supplementary file 1 — Supplementary Material 1. [file 590_2025_4598_MOESM1_ESM.docx]

Supplementary Data

Table SI: Clinical Data

|  | **PF, n=51** | **ARPF, n=38** | **P** |
| --- | --- | --- | --- |
| Total Operative Time, minutes | 221.2 ± 60.8 | 398.9 ± 86.3 | <0.0001* |
| Total Estimated Blood Loss, ml | 690.2 ± 523.5 | 965.3 ± 446.3 | 0.0091* |
| LOS, days | 8.6 ± 6.6 | 14.5 ± 8.9 | 0.001* |
| Complications, n (%) | 4 (7.8%) | 10 (26.3%) | 0.8268† |
| Infection | 1 | 2 |  |
| Instrumentation Failure | 1 | 3 |  |
| Pulmonary Complication | 0 | 3 |  |
| Neurological Injury | 1 | 1 |  |
| Durotomy | 1 | 1 |  |
| Return to theatre, n (%) | 0 (0%) | 1 (2.6%) | 0.427~ |
| *Welch's t test † Chi-squared test ~ Fisher's exact test | | | |
